# Synergistic Prognostic Value of Epicardial Fat Volume and Triglyceride-Glucose Index in Heart Failure Patients With Glycemic Dysregulation

**DOI:** 10.31083/RCM49554

**Published:** 2026-07-14

**Authors:** Ziqi Chen, Ying Yang, Huiwen Chen, Qilin Li, Haifeng Zhang, Iokfai Cheang, Xinli Li

**Affiliations:** ^1^State Key Laboratory for Innovation and Transformation of Luobing Theory, Department of Cardiology, The First Affiliated Hospital of Nanjing Medical University, Jiangsu Province Hospital, 210029 Nanjing, Jiangsu, China; ^2^Department of Cardiology, The Affiliated Suzhou Hospital of Nanjing Medical University, Suzhou Municipal Hospital, Gusu School, 215002 Suzhou, Jiangsu, China

**Keywords:** chronic heart failure, epicardial fat volume, triglyceride-glucose index, glycemic dysregulation, prognostic modeling

## Abstract

**Background:**

Glycemic abnormalities are highly prevalent in chronic heart failure (CHF) and exacerbate adverse cardiac remodeling. Epicardial fat volume (EFV) and the triglyceride-glucose (TyG) index may both reflect metabolic-cardiovascular interactions, but their combined prognostic utility in CHF patients with dysglycemia remains unexplored.

**Methods:**

We analyzed 516 CHF patients stratified by glycemic status: normal glucose (n = 230), prediabetes (n = 174), and diabetes (n = 112). Baseline characteristics, echocardiography (TTE), and cardiac magnetic resonance (CMR) data were assessed. The primary endpoint was defined as a composite of heart-failure hospitalization or all-cause mortality and was evaluated during a follow-up period of up to 3 years. Associations between EFV, TyG index, and primary endpoint were evaluated using stratified, joint, and incremental prognostic models.

**Results:**

Diabetic CHF patients were generally older, had higher smoking rates, worse renal function, and poorer nutritional status. CMR and TTE revealed more severe cardiac structural and functional impairments in dysglycemic groups, including reduced left ventricular ejection fraction (LVEF) and elevated EFV. EFV predicted primary endpoint with increasing accuracy across glycemic strata (AUC: 0.631–0.711 at 1–3 years), with the strongest predictive performance in diabetes. A significant correlation between TyG index and EFV was observed in diabetic patients (r = 0.24, *p* = 0.00021). Joint EFV (cutoff: 74.68 mL/m²) and TyG (median: 8.48) analysis showed that high TyG alone or combined high EFV/TyG conferred the highest the risk of primary endpoint (*p* for trend < 0.001), while isolated EFV elevation did not. Incorporating EFV into TyG-based models significantly improved prediction (AUC increased from 0.698 to 0.729 at 1 year). Net reclassification and integrated discrimination improvements confirmed the incremental prognostic value of EFV, particularly in diabetes and over longer follow-up.

**Conclusion:**

In CHF patients with glycemic dysregulation, EFV and TyG index synergistically enhance clinical endpoint prediction, with EFV providing significant incremental prognostic value beyond TyG. These metrics may improve risk stratification and personalized management in comorbid heart failure and dysglycemia.

## 1. Introduction

Chronic heart failure (CHF) represents a major and growing public health burden worldwide, characterized by high rates of morbidity, mortality, and recurrent hospitalization [1,2]. The pathophysiology of heart failure (HF) is complex and intertwined with numerous comorbid conditions, among which metabolic dysregulation plays a pivotal role. Abnormal energy substrate utilization, insulin resistance, and chronic inflammatory states contribute significantly to myocardial remodeling, systolic and diastolic dysfunction, and the overall progression of the HF syndrome [3,4].

Glycemic abnormalities, encompassing the spectrum from insulin resistance and prediabetes to overt type 2 diabetes mellitus (T2DM), are exceptionally prevalent in the HF population and are recognized as potent drivers of adverse outcomes [5,6]. Dysglycemia exacerbates HF through multiple mechanisms, including promotion of endothelial dysfunction, accumulation of advanced glycation end-products, and induction of lipotoxicity, which collectively accelerate adverse cardiac remodeling and increase the risk of clinical endpoint [7,8]. Despite the clear association, the optimal tools for risk stratification within this high-risk comorbid population remain limited, necessitating the exploration of novel biomarkers that reflect the underlying metabolic-cardiac interface.

Two promising markers have emerged in this context: epicardial fat volume (EFV) and the triglyceride-glucose (TyG) index. Epicardial adipose tissue, quantified by EFV via cardiac magnetic resonance (CMR) or computed tomography, is a metabolically active organ that directly surrounds the coronary arteries and myocardium. Due to its anatomic proximity and lack of a fascial barrier, it can locally secrete pro-inflammatory cytokines and free fatty acids, directly contributing to coronary atherosclerosis, myocardial fibrosis, and ventricular dysfunction [9,10]. The TyG index is a simple and reliable surrogate marker of insulin resistance [11]. It has been demonstrated to predict new-onset HF and adverse prognosis in cardiovascular diseases [12,13]. Both EFV and the TyG index are elevated in settings of glycemic dysregulation and are independently associated with worse cardiovascular outcomes.

However, their interplay and combined prognostic utility specifically in patients with established HF and comorbid glycemic dysregulation are not well understood. It is plausible that these markers capture complementary pathophysiological pathways of systemic insulin resistance (TyG index) and local paracrine cardiac toxicity (EFV) could synergistically offer superior risk stratification.

Therefore, the primary objective of this study was to investigate the individual and synergistic prognostic value of EFV and the TyG index for predicting endpoint in the cohort of chronic HF patients, stratified by glycemic status. We hypothesized that the combination of high EFV and a high TyG index would identify a subgroup of HF patients with glycemic dysregulation at the highest risk for endpoint and that EFV would provide incremental prognostic value beyond traditional risk factors and the TyG index alone.

## 2. Methods

### 2.1 Study Design

This retrospective single-center cohort study enrolled 516 patients with HF who underwent CMR evaluation between January 2018 and December 2020 in the First Affiliated Hospital of Nanjing Medical University (Nanjing, China). Participants with age over 18 years old and less than 85 years were included in our design. The diagnosis of HF were conducted according to the Chinese guideline for diagnosis and treatment of heart failure [14]. The evaluation and treatment of HF were performed in accordance with clinical history, physical assessment, laboratory investigations, cardiac imaging, and functional testing results.

The study protocol was approved by the independent institutional ethics committee of the First Affiliated Hospital of Nanjing Medical University in accordance with the Declaration of Helsinki (Approval No. 2022-SR-052). All participants provided written informed consent prior to enrollment.

### 2.2 Definition of Covariates

Demographic information, physical examination reports, New York Heart Association (NYHA) functional class, electrocardiographic (ECG) results, laboratory tests, clinical characteristics, comorbidities, and underlying etiology of CHF were extracted from the hospital’s electronic medical record system. All laboratory analyses were performed in the central laboratory of our institution.

We conducted transthoracic echocardiography (TTE) with a Vivid E9 ultrasound system (GE Medical System, USA), and left ventricular ejection fraction (LVEF) was measured following the modified Simpson’s method.

Diabetes was defined as fasting blood glucose (FBG) >126 mg/dL, HbA1c ≥6.5%, medical diagnosis, or use of glucose-lowering medication. While Prediabetes was clarified as FBG between 100 and 125 mg/dL or HbA1c between 5.7% and 6.4%, without previous diagnosed as diabetes or consuming any hypoglycemics agents [15]. Meanwhile, hypertension was characterized as systolic blood pressure (SBP) ≥140 mmHg, diastolic blood pressure (DBP) ≥90 mmHg, antihypertensive medication use, or clinical diagnoses [16].

Especially, the triglyceride-glucose index (TyG), a marker of insulin resistance, was calculated with the formula: TyG = ln[fasting triglycerides (mg/dL) × fasting glucose (mg/dL)/2].

### 2.3 Measurement of Outcomes

The primary endpoint was a composite outcome of heart-failure hospitalization or all-cause mortality during follow-up. Heart-failure hospitalization was defined as admission requiring treatment for worsening heart failure symptoms. Information on endpoints and patients’ status were obtained via telephone follow-up and/or outpatient visits, with confirmation from the patient’s family or treating physician.

### 2.4 CMR and Epicardial Fat Quantification (EFV)

Participants underwent CMR imaging in the supine position using a 3T scanner (MAGNETOM Skyra, Siemens Healthcare, Germany) with an 18-channel phased-array body coil anteriorly and a spine coil posteriorly. All acquisitions were collected under ECG-gated breath-hold conditions. The imaging protocol included balanced steady-state free-precession (b-SSFP) cine sequences in three long-axis views (two-, three-, and four-chamber) and a contiguous short-axis stack covering the left ventricle from base to apex (TR/TE 3.4/1.4 ms, flip angle 47°, field of view 360 × 360 mm^2^, matrix 208 × 188, voxel size 1.6 × 1.6 × 8.0 mm^3^, slice thickness 8 mm with 2-mm gap, temporal resolution 34 ms). Late gadolinium enhancement (LGE) imaging was performed 10–15 minutes after intravenous administration of gadolinium-DTPA (0.2 mmol/kg, Magnevist, Schering, Berlin, Germany) utilizing a phase-sensitive inversion recovery (PSIR) sequence.

All images were analyzed offline using CVI 42 software (Circle Cardiovascular Imaging Inc., Calgary, Canada) by two independent clinicians, with a third cardiovascular radiologist adjudicating in cases of disagreement. End-diastolic volumes, stroke volumes, LGE, and T1 mapping values were recorded according to guidelines. Extracellular volume (ECV) was calculated from native and post-contrast T1 measurements at the septal mid-ventricular short-axis level.

EFV was measured based on established methods [17,18]. Endocardial and epicardial borders of both ventricles were manually traced on end-diastolic short-axis slices from base to apex. The mitral valve annulus was applied to distinguish atrial from ventricular epicardial adipose tissue. Ventricular epicardial adipose tissue was identified as fat surrounding the ventricles from the mitral valve to the apex. Coronary arteries were manually excluded. EFV was then indexed to body surface area (BSA). Subsequently, in order to confirm that the primary representative value as adipose tissue of measured EFV, T1 mapping was operated at the mid-ventricular level to assess T1 relaxation times of myocardium, epicardial fat, subcutaneous fat, and blood pool. Comparable T1 values between epicardial and subcutaneous fat ensured that the volume measurements reflected adipose tissue rather than fluids.

### 2.5 Statistical Analyses

Continuous variables were presented as mean ± standard deviation (SD) for normally distributed data or median (interquartile range, IQR) for non-normally distributed data. Categorical variables were expressed as counts (percentages). Group comparisons were performed using Student’s *t*-test or Mann–Whitney U test for continuous variables and the chi-square (χ^2^) test for categorical variables.

Internal validation of the epicardial fat volume (EFV) cut-off derived from receiver operating characteristic curves (ROC) was performed using bootstrap resampling (1000 iterations) to assess the stability of the estimated threshold. Kaplan–Meier (KM) curves were applied to evaluate differences in the incidence of the primary endpoint across subgroups. Cox proportional hazards regression models were used to estimate hazard ratios (HR) and 95% confidence intervals (CI). Covariates included in the multivariable models were selected based on clinical relevance and prior evidence regarding prognostic factors in HF. The fully adjusted model included age, gender, SBP, DBP, red blood cell distribution width (RDW), total bilirubin (TBIL), blood urea nitrogen (BUN), estimated glomerular filtration rate (eGFR), uric acid (UA), N-terminal pro-brain natriuretic peptide (NT-proBNP), left atrial diameter (LAD), LVEF, left ventricular end-systolic diameter (LVDs), left ventricular end-diastolic diameter (LVDd), atrial fibrillation (AF), NYHA class.

Collinearity among covariates was evaluated using variance inflation factors (VIF). All variables included in the analyses were complete, and no missing data were observed. Model performance and incremental predictive ability were assessed using time-dependent area under the curve (AUC), C-index, net reclassification improvement (NRI), and integrated discrimination improvement (IDI).

## 3. Results

### 3.1 Baseline Characteristics

According to baseline information, participants were categorized into three groups: normal glucose (n = 230), prediabetes (n = 174), and diabetes (n = 112). Baseline demographic and clinical characteristics across different subgroups were summarized in Table 1. Participants diagnosed as diabetes were more likely to be older, smoker and suffered impaired renal function and poorer nutritional status. However, no significant differences were observed in control of blood pressure and lipids. Both TTE and CMR data consistently indicated that patients with glycemic dysregulation exhibited compromised cardiac function and structural abnormalities, particularly reduced LVEF and elevated EFV. Meanwhile, significantly decreased incidence ratio of primary endpoint was observed in normal individuals (*p* = 0.024).

**Table 1.** **Baseline characteristics**.

Variables		Normal (n = 230)	Prediabetes (n = 174)	Diabetes (n = 112)	*p*
Age, (years)		46.45 ± 16.75	52.34 ± 14.54	56.28 ± 15.36	<0.001
BMI, (kg/m²)		24.01 ± 4.72	24.86 ± 4.03	25.00 ± 3.55	0.054
SBP, (mmHg)		121.15 ± 16.08	118.91 ± 18.45	123.62 ± 17.12	0.075
DBP, (mmHg)		75.02 ± 12.23	75.97 ± 12.69	76.12 ± 12.86	0.655
RDW, (%)		12.99 ± 1.49	13.61 ± 1.60	13.58 ± 1.79	<0.001
ALB, (g/L)		38.64 ± 3.86	37.79 ± 4.05	37.24 ± 4.33	0.007
TC, (mmol/L)		4.04 ± 0.98	4.22 ± 1.07	4.09 ± 1.20	0.220
TG, (mmol/L)		1.40 ± 0.94	1.40 ± 0.75	1.63 ± 1.27	0.089
HDL-c, (mmol/L)		1.11 ± 0.92	1.03 ± 0.27	0.98 ± 0.24	0.143
LDL-c, (mmol/L)		2.45 ± 0.72	2.63 ± 0.80	2.51 ± 0.87	0.069
TBIL, (μmol/L)		13.66 ± 6.89	14.92 ± 9.02	16.92 ± 14.60	0.015
BUN, (mmol/L)		6.27 ± 3.58	6.68 ± 4.68	7.10 ± 2.70	0.159
eGFR, (mL/min/1.73 m^2^)		103.61 ± 40.96	98.34 ± 26.89	89.17 ± 30.47	0.001
Glucose, (mmol/L)		4.50 ± 0.55	4.73 ± 0.70	6.64 ± 2.82	<0.001
HbA1c, (%)		5.31 ± 0.27	5.93 ± 0.22	7.36 ± 1.43	<0.001
TyG		8.34 ± 0.60	8.45 ± 0.50	8.81 ± 0.71	<0.001
UA, (μmol/L)		373.59 ± 118.51	429.91 ± 139.89	425.42 ± 127.66	<0.001
LAD, (mm)		39.05 ± 7.82	43.94 ± 8.12	44.19 ± 7.67	<0.001
LVDd, (mm)		54.36 ± 10.30	60.70 ± 11.89	60.87 ± 11.64	<0.001
LVDs, (mm)		40.28 ± 12.42	48.11 ± 13.61	48.65 ± 14.32	<0.001
LVEF, (%)		51.72 ± 14.41	41.56 ± 14.33	40.94 ± 14.53	<0.001
SV, (mL)		78.40 ± 25.82	70.10 ± 24.22	67.58 ± 23.60	<0.001
CO, (L/min)		5.18 ± 1.70	4.88 ± 1.50	4.72 ± 1.49	0.026
LVM, (g)		157.79 ± 60.56	181.48 ± 71.27	183.16 ± 55.90	<0.001
LVMED, (g)		156.47 ± 60.08	180.81 ± 72.07	183.89 ± 58.04	<0.001
EDV, (mL)		201.31 ± 99.54	253.11 ± 131.59	250.77 ± 117.42	<0.001
ESV, (mL)		120.74 ± 95.56	183.31 ± 124.97	183.12 ± 114.41	<0.001
T1, (ms)		1367.62 ± 105.69	1384.04 ± 118.65	1364.55 ± 122.02	0.258
ECV, (%)		34.02 ± 13.38	34.46 ± 11.36	34.66 ± 11.99	0.889
EFV, (mL/m²)		52.87 ± 23.52	63.27 ± 25.97	66.64 ± 26.18	<0.001
NT-proBNP, (pg/mL)		555.40 (173.75, 1387.50)	1105.00 (369.40, 2738.20)	1393.00 (355.78, 3287.50)	<0.001
ALT, (U/L)		22.05 (15.03, 31.90)	25.35 (17.33, 45.85)	26.85 (18.20, 45.20)	<0.001
AST, (U/L)		21.85 (18.00, 29.62)	25.50 (19.60, 35.85)	26.40 (19.82, 36.78)	<0.001
D-dimer, (mg/L FEU)		0.21 (0.12, 0.52)	0.29 (0.16, 0.69)	0.41 (0.17, 0.92)	<0.001
Gender					0.189
	Male	79 (34.35)	47 (27.01)	30 (26.79)	
	Female	151 (65.65)	127 (72.99)	82 (73.21)	
Primary endpoint, n (%)					0.024
	No	183 (79.57)	120 (68.97)	77 (68.75)	
	Yes	47 (20.43)	54 (31.03)	35 (31.25)	
NYHA, n (%)					<0.001
	1	69 (30.00)	32 (18.39)	11 (9.82)	
	2	93 (40.43)	55 (31.61)	44 (39.29)	
	3	56 (24.35)	73 (41.95)	39 (34.82)	
	4	12 (5.22)	14 (8.05)	18 (16.07)	
HBP, n (%)					<0.001
	No	170 (73.91)	114 (65.52)	57 (50.89)	
	Yes	60 (26.09)	60 (34.48)	55 (49.11)	
CAD, n (%)					0.002
	No	198 (86.09)	143 (82.18)	79 (70.54)	
	Yes	32 (13.91)	31 (17.82)	33 (29.46)	
AF, n (%)					0.001
	No	202 (87.83)	128 (73.56)	92 (82.14)	
	Yes	28 (12.17)	46 (26.44)	20 (17.86)	
Smoke, n (%)					0.011
	No	176 (76.52)	112 (64.37)	72 (64.29)	
	Yes	54 (23.48)	62 (35.63)	40 (35.71)	
Drink, n (%)					0.479
	No	176 (76.52)	124 (71.26)	82 (73.21)	
	Yes	54 (23.48)	50 (28.74)	30 (26.79)	

Abbreviations: BMI, body mass index; NT-proBNP, N-terminal pro–B-type natriuretic peptide; SBP, systolic blood pressure; DBP, diastolic blood pressure; RDW, red cell distribution width; ALB, albumin; TC, total cholesterol; TG, triglycerides; HDL-c, high-density lipoprotein cholesterol; LDL-c, low-density lipoprotein cholesterol; TBIL, total bilirubin; ALT, alanine aminotransferase; AST, aspartate aminotransferase; BUN, blood urea nitrogen; eGFR, estimated glomerular filtration rate; Glucose, fasting blood glucose; HbA1c, glycated hemoglobin; TyG, triglyceride–glucose index; UA, uric acid; D-dimer, D-dimer; LAD, left atrial diameter; LVDd, left ventricular end-diastolic diameter; LVDs, left ventricular end-systolic diameter; LVEF, left ventricular ejection fraction; SV, stroke volume; CO, cardiac output; LVM, left ventricular mass; LVMED, left ventricular mass indexed to end-diastolic volume; EDV, end-diastolic volume; ESV, end-systolic volume; T1, native T1 relaxation time; ECV, extracellular volume fraction; EFV, epicardial fat volume; NYHA, New York Heart Association functional class; HBP, hypertension; CAD, coronary artery disease; AF, atrial fibrillation. Values are presented as mean ± standard deviation (SD) for normally distributed data, median (interquartile range, IQR) for skewed variables, and number (percentage) for categorical variables.

### 3.2 Stratified Analyses

**Fig. 1.**
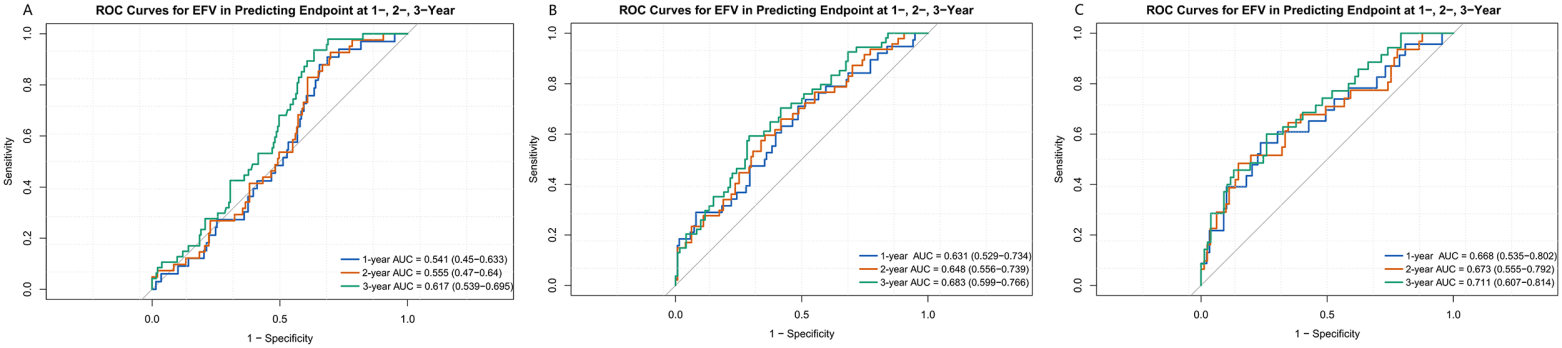
**Time-dependent ROC analyses for 1-, 2-, and 3-year primary endpoint of epicardial fat volume in different subgroups of heart failure patients**. (A) Normal; (B) Prediabetes; (C) Diabetes. Abbreviations: ROC, receiver operating characteristic curve; AUC, area under curve.

**Fig. 2.**
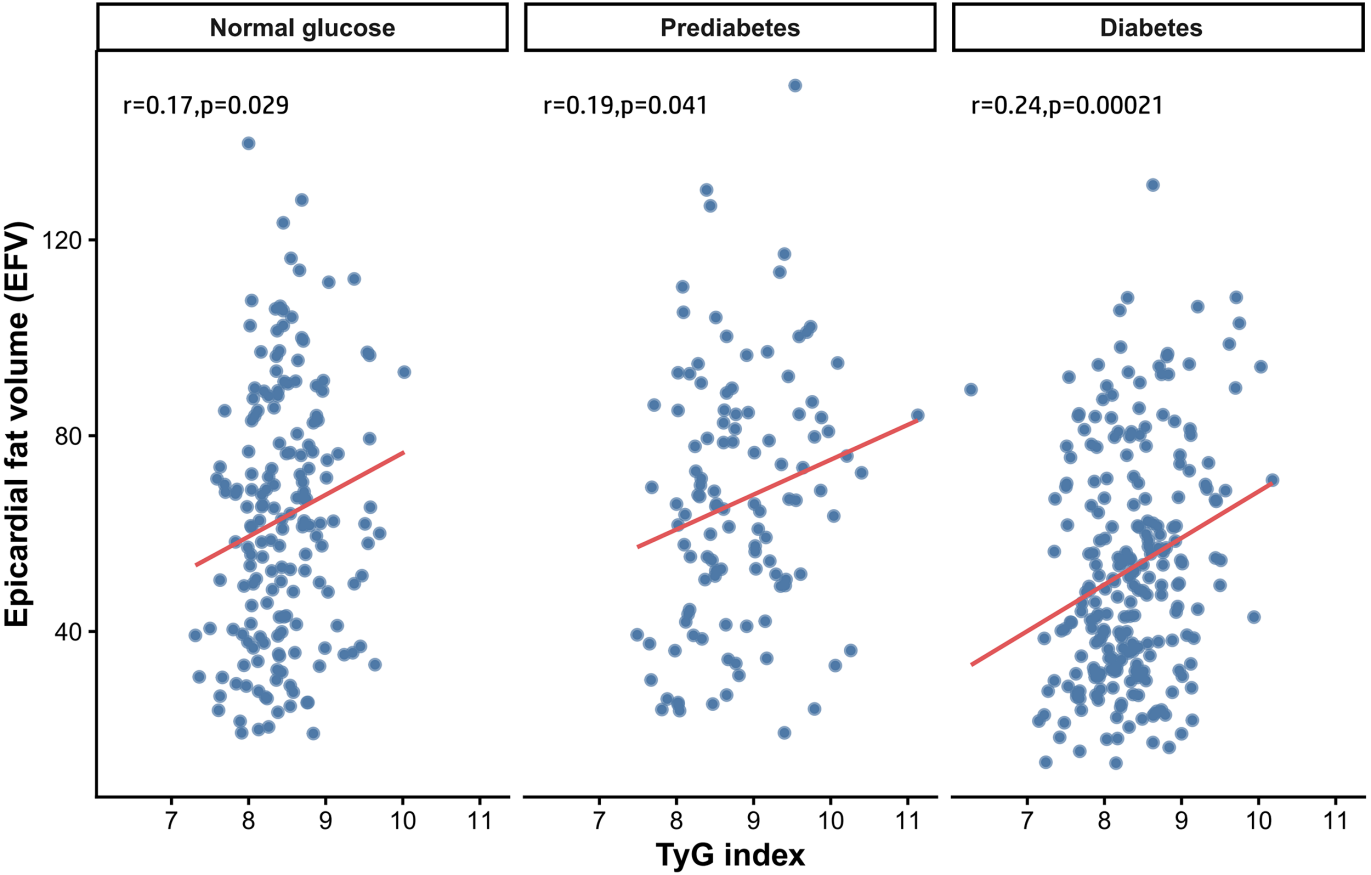
**The association between TyG and epicardial fat volume**.

Stratified analyses by glycemic status were presented in Fig. 1. Consistent with our previous conclusions among the overall cohort, EFV indicated modest association with the occurrence of primary endpoint in patients with normal glucose metabolism. However, the improved prognostic performance of EFV varied according to glycemic status with AUCs achieving 0.631, 0.648, and 0.683 for patients with prediabetes, AUCs of 0.668, 0.673, and 0.711 for patients with diabetes at 1-, 2-, and 3-year respectively. Similarly, Cox regression of Table 2 suggested only correlation between EFV and primary endpoint among patients with normal glycemia faded into insignificance at different timepoints (HR = 1.00, 95% CI: 0.99–1.02, *p* = 0.793 for 1-year timepoint; HR = 1.00, 95% CI: 0.99–1.02 for 2-years timepoint, *p* = 0.661; HR = 0.99, 95% CI: 0.98–1.00, *p* = 0.453 for 3-years timepoint). Meanwhile, we also observed the strongest positive relation between the TyG index and EFV in patients with diabetes (r = 0.24, *p* = 0.00021, Fig. 2).

**Table 2.** **Cox regression across subgroups stratified by glycemic status**.

	Subgroups		
	Normal (n = 230)	Prediabetes (n = 174)	Diabetes (n = 112)
HR for 1-year (95% CI)	1.00 (0.99, 1.02)	1.02 (1.01, 1.03)	1.03 (1.01, 1.05)
*p*	0.793	0.042	0.002
HR for 2-year (95% CI)	1.00 (0.99, 1.02)	1.02 (1.01, 1.03)	1.02 (1.01, 1.03)
*p*	0.661	0.032	0.024
HR for 3-year (95% CI)	0.99 (0.98, 1.00)	1.01 (1.00, 1.02)	1.01 (1.00, 1.02)
*p*	0.453	0.036	0.012

Adjusted by: age, gender, systolic blood pressure, diastolic blood pressure, red blood cell distribution width, total bilirubin, blood urea nitrogen, estimated glomerular filtration rate, uric acid, N-terminal pro-brain natriuretic peptide, left atrial diameter, left ventricular end-diastolic diameter, left ventricular end-systolic diameter, left ventricular ejection fraction, atrial fibrillation, New York Heart Association class, extracellular volume. Abbreviations: HR, hazard ratio; CI, confidence interval.

### 3.3 Joint Analyses of EFV and TyG With Primary Endpoint

Furthermore, we applied ROC to identify the cutoff value of EFV as 74.68 mL/m² with a 95% confidence interval of 46.19–111.54 for predicting primary endpoint among patients with glycemic disorder (n = 286). Combined with calculated median value of TyG as 8.48, we divided the entire cohort as four groups (High/Low EFV with High/Low TyG).

**Fig. 3.**
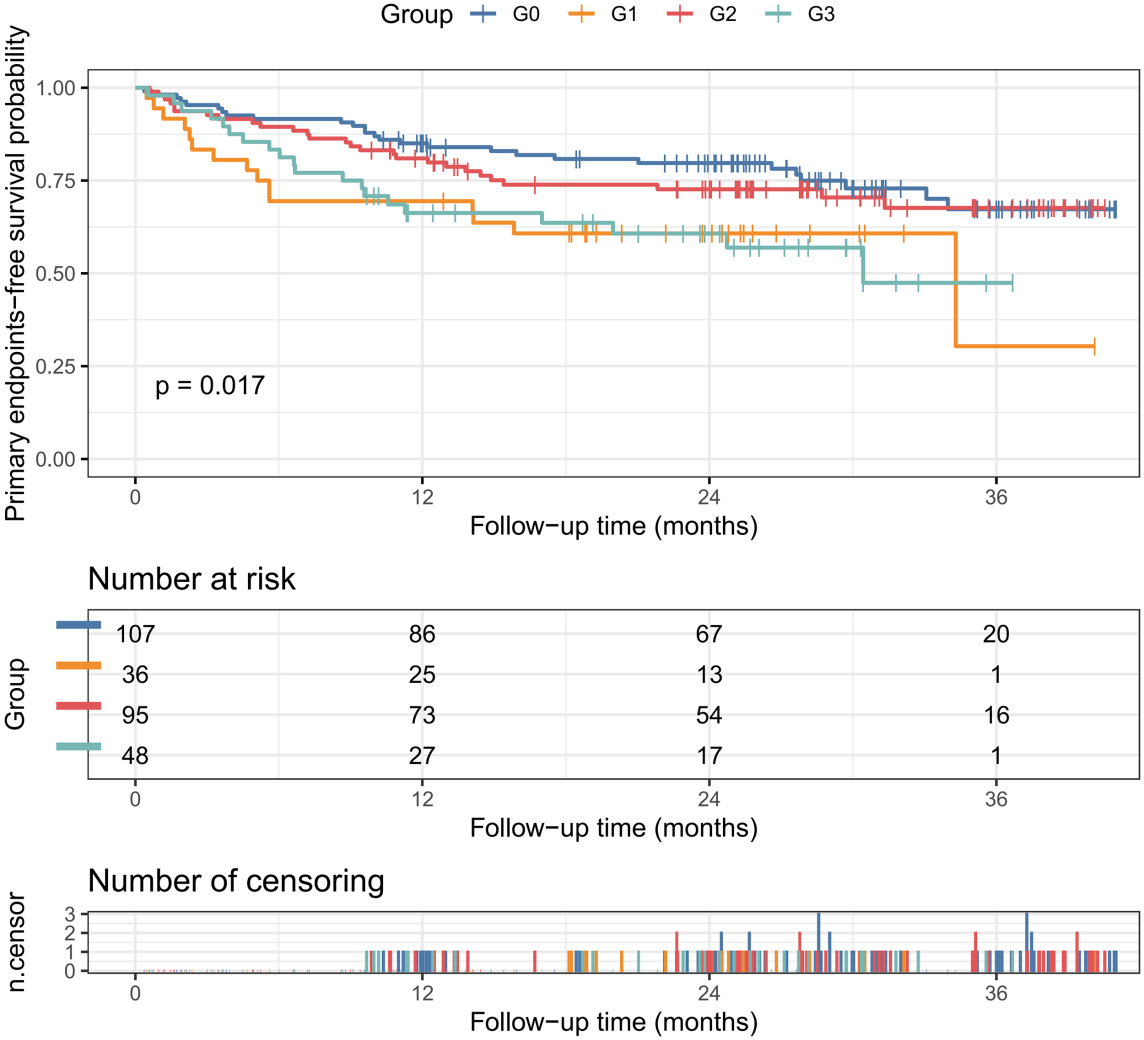
**Kaplan-Meier survival curve of different groups in predicting primary endpoint of heart failure patients with glycemic disorder**. G0: patients with EFV < cutoff and TyG < median; G1: patients with EFV < cutoff and TyG ≥ median; G2: patients with EFV ≥ cutoff and TyG < median; G3: patients with EFV ≥ cutoff and TyG ≥ median.

As summarized in Fig. 3 and Table 3, when patients were stratified according to combined EFV and TyG categories, those with elevated TyG alone (Group 1) and those with concomitantly elevated EFV and TyG (Group 3) exhibited a significantly higher risk of primary endpoint compared to the reference group in both unadjusted and multivariable-adjusted models. In contrast, elevated EFV in the absence of high TyG (Group 2) was not independently associated with adverse endpoints (HR = 1.18, 95% CI: 0.69–2.01 for model 1, *p* = 0.326; HR = 1.47, 95% CI: 0.84–2.58 for model 2, *p* = 0.239). The overall trend across the four groups was statistically significant in both models (*p* for trend <0.001), indicating that EFV offered incremental prognostic information beyond TyG among patients with glycemic dysregulation.

**Table 3.** **Cox regression of different groups in predicting primary endpoint of heart failure patients with glycemic disorder**.

Models	Groups				*p* for trend
	Group 0	Group 1	Group 2	Group 3	
Model 1	Reference	2.14 (1.14, 4.06)	1.18 (0.69, 2.01)	2.10 (1.17, 3.76)	<0.001
Model 2	Reference	2.04 (1.06, 3.95)	1.47 (0.84, 2.58)	2.09 (1.13, 3.85)	<0.001

Model 1: Unadjusted; Model 2: Adjusted by age, gender, systolic blood pressure, diastolic blood pressure, red blood cell distribution width, total bilirubin, blood urea nitrogen, estimated glomerular filtration rate, uric acid, N-terminal pro-brain natriuretic peptide, left atrial diameter, left ventricular end-diastolic diameter, left ventricular end-systolic diameter, left ventricular ejection fraction, atrial fibrillation, New York Heart Association class. G0: patients with EFV < cutoff and TyG < median; G1: patients with EFV < cutoff and TyG ≥ median; G2: patients with EFV ≥ cutoff and TyG < median; G3: patients with EFV ≥ cutoff and TyG ≥ median.

### 3.4 Prognostic Models and Incremental Efficacy

VIF analyses of including variables confirmed the absence of severe collinearity with all VIF values <5 (Table 4), ensuring the stability of constructed models.

**Table 4.** **Variance inflation factor analyses of selected covariates**.

Variables	VIF
Age	1.669285
Gender	1.225420
BMI	1.596397
HBP	1.305942
eGFR	1.500354
UA	1.314859
NT-proBNP	1.448500
NYHA	1.640213
AF	1.172935
CAD	1.140519
TyG	1.153706
LVEF	1.518661
EFV	1.225672
Smoke	1.804316
Drink	1.773341

**Fig. 4.**
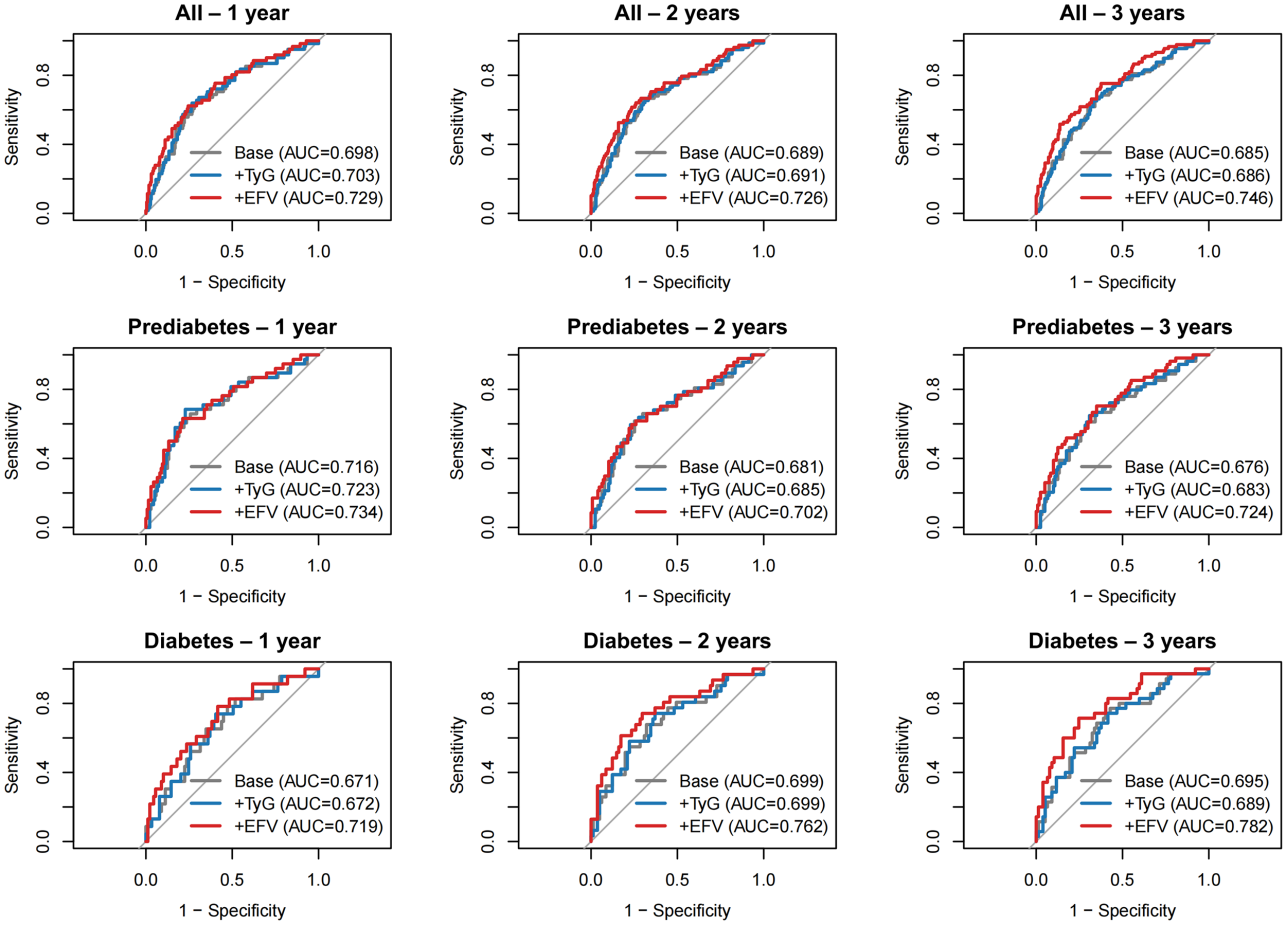
**Time-dependent ROC analyses for 1-, 2-, and 3-year primary endpoint of epicardial fat volume in different subgroups of heart failure patients**.

Among dysglycemic individuals, stepwise modifications of the base model by incorporation of TyG and EFV caused incremental improvements in model performance for predicting primary endpoint. Time-dependent ROC analyses demonstrated modest enhancement in the AUC after the addition of TyG, whereas further inclusion of EFV resulted in significant elevated efficiency, particularly at longer follow-up durations. For example, in the overall population, the AUC increased from 0.698 in the base model to 0.703 after inclusion of TyG and to 0.729 after additional adjustment for EFV at 1 year. Similar patterns were observed in individuals with prediabetes and diabetes, with the highest AUC values were constantly achieved by the TyG + EFV model (Fig. 4, Table 5). Time-specific calibration slope analyses demonstrated slopes approaching unity across all time points, particularly at 3 years, indicating stable long-term performance and robust calibration of the combined TyG + EFV model across glycemic subgroups (Table 6). Consistent with the AUC analyses, discrimination assessed by the C-index verified incremental variations following sequential model adjustment (Table 7).

**Table 5.** **AUC of different models adjusted by triglyceride–glucose index (TyG) and epicardial fat volume (EFV) for the incidence of primary endpoint at 1-, 2-, 3-year**.

Group	Time	Model	AUC
All	1 year	Base	0.698 (0.622–0.774)
All	1 year	Base+TyG	0.703 (0.628–0.779)
All	1 year	Base+TyG+EFV	0.729 (0.655–0.803)
All	2 years	Base	0.689 (0.618–0.760)
All	2 years	Base+TyG	0.691 (0.620–0.762)
All	2 years	Base+TyG+EFV	0.726 (0.657–0.795)
All	3 years	Base	0.685 (0.618–0.751)
All	3 years	Base+TyG	0.686 (0.620–0.753)
All	3 years	Base+TyG+EFV	0.746 (0.685–0.808)
Prediabetes	1 year	Base	0.716 (0.618–0.814)
Prediabetes	1 year	Base+TyG	0.723 (0.626–0.821)
Prediabetes	1 year	Base+TyG+EFV	0.734 (0.640–0.829)
Prediabetes	2 years	Base	0.681 (0.587–0.775)
Prediabetes	2 years	Base+TyG	0.685 (0.592–0.778)
Prediabetes	2 years	Base+TyG+EFV	0.702 (0.610–0.794)
Prediabetes	3 years	Base	0.676 (0.588–0.763)
Prediabetes	3 years	Base+TyG	0.683 (0.596–0.769)
Prediabetes	3 years	Base+TyG+EFV	0.724 (0.642–0.806)
Diabetes	1 year	Base	0.671 (0.546–0.795)
Diabetes	1 year	Base+TyG	0.672 (0.548–0.797)
Diabetes	1 year	Base+TyG+EFV	0.719 (0.598–0.840)
Diabetes	2 years	Base	0.699 (0.587–0.811)
Diabetes	2 years	Base+TyG	0.699 (0.587–0.810)
Diabetes	2 years	Base+TyG+EFV	0.762 (0.658–0.866)
Diabetes	3 years	Base	0.695 (0.589–0.801)
Diabetes	3 years	Base+TyG	0.689 (0.583–0.796)
Diabetes	3 years	Base+TyG+EFV	0.782 (0.690–0.875)

**Table 6.** **Time-specific calibration slope of models adjusted by TyG and EFV for the incidence of primary endpoint at 1-, 2-, 3-year**.

Group	Time	Slope	Intercept
All	1 year	1.321	–2.127
All	2 years	1.132	–1.602
All	3 years	1.107	–1.347
Prediabetes	1 year	1.384	–2.102
Prediabetes	2 years	1.027	–1.509
Prediabetes	3 years	1.01	–1.234
Diabetes	1 year	1.247	–2.178
Diabetes	2 years	1.313	–1.778
Diabetes	3 years	1.291	–1.572

**Table 7.** **C-index of different models adjusted by TyG and EFV for the incidence of primary endpoint**.

Group	Model	Cindex
All	Base	0.673 (0.613–0.733)
All	Base+TyG	0.676 (0.616–0.737)
All	Base+TyG+EFV	0.684 (0.624–0.745)
Prediabetes	Base	0.676 (0.598–0.754)
Prediabetes	Base+TyG	0.681 (0.604–0.758)
Prediabetes	Base+TyG+EFV	0.694 (0.621–0.768)
Diabetes	Base	0.684 (0.585–0.784)
Diabetes	Base+TyG	0.717 (0.626–0.809)
Diabetes	Base+TyG+EFV	0.757 (0.676–0.838)

**Fig. 5.**
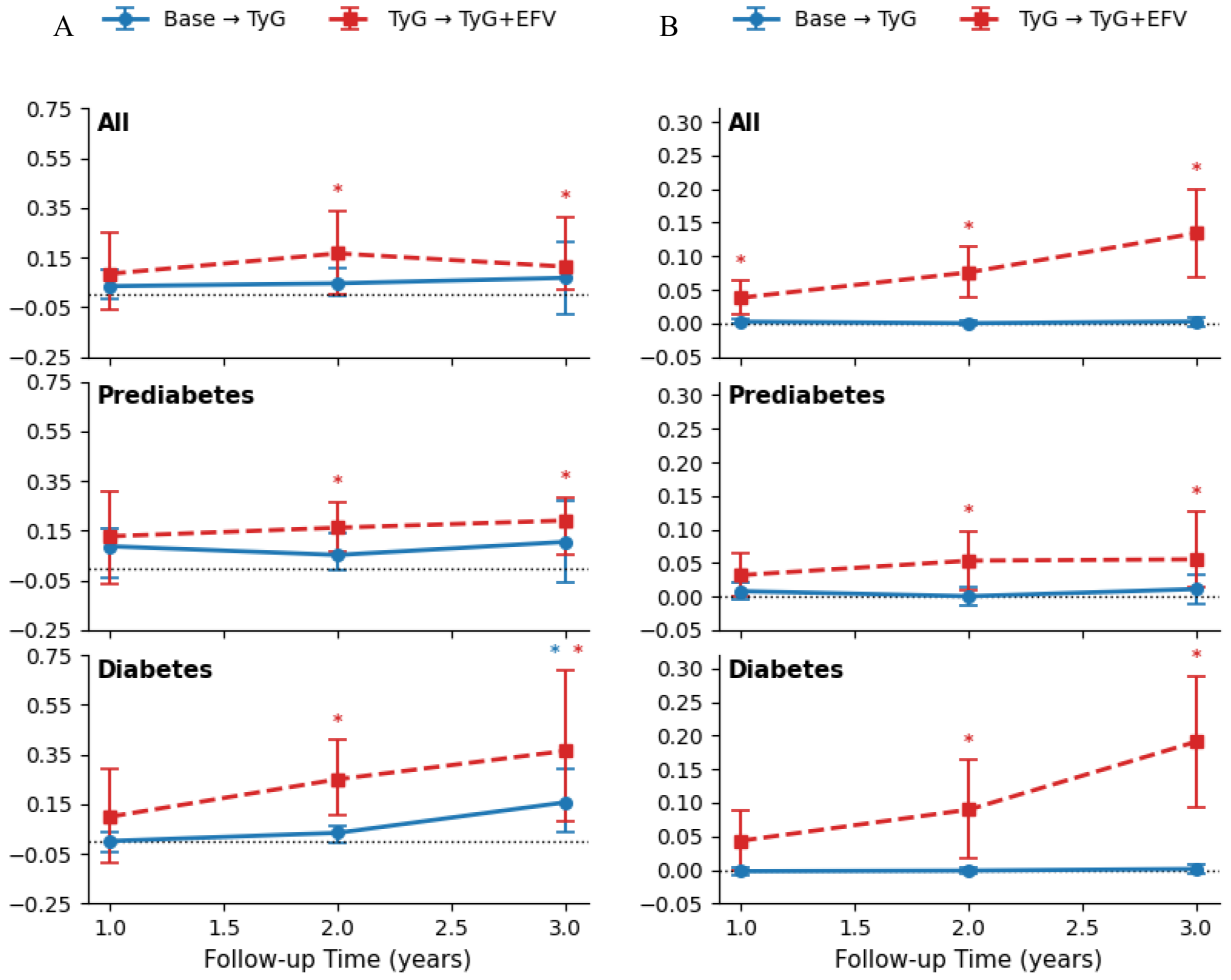
**Incremental reclassification and discrimination improvement after adding EFV to TyG-based prognostic models**. (A) NRI of different models; (B) IDI of different models. Abbreviations: NRI, net reclassification improvement index; IDI, integrated discrimination improvement.

NRI and IDI analyses provided complementary evidence for the incremental prognostic value of EFV. Integration of EFV into the TyG based model yielded further and substantial reclassification gains, particularly at longer follow-up periods and among participants with diabetes (Fig. 5, Table 8). Moreover, IDI analyses delivered that inclusion of EFV prompted the model discrimination especially in prediabetes and diabetes subgroups, while the addition of TyG alone was associated with relatively smaller changes in discrimination (Fig. 5, Table 9).

**Table 8.** **NRI of different models adjusted by TyG and EFV for the incidence of primary endpoint at 1-, 2-, 3-year**.

Group	Time	Comparison	NRI_total	NRI_Lower	NRI_Upper	*p*
All	1 year	Base → TyG	0.0348	–0.0196	0.1013	0.252
All	1 year	TyG → TyG+EFV	0.0848	–0.0595	0.2495	0.274
All	2 years	Base → TyG	0.0462	–0.0046	0.1052	0.084
All	2 years	TyG → TyG+EFV	0.1660	0.0018	0.3366	0.039
All	3 years	Base → TyG	0.0684	–0.0803	0.2148	0.402
All	3 years	TyG → TyG+EFV	0.1133	0.0192	0.3139	0.033
Prediabetes	1 year	Base → TyG	0.0877	–0.0420	0.1597	0.518
Prediabetes	1 year	TyG → TyG+EFV	0.1279	–0.0666	0.3096	0.214
Prediabetes	2 years	Base → TyG	0.0527	–0.0084	0.1419	0.637
Prediabetes	2 years	TyG → TyG+EFV	0.1626	0.0641	0.2671	0.020
Prediabetes	3 years	Base → TyG	0.1055	–0.0577	0.2682	0.825
Prediabetes	3 years	TyG → TyG+EFV	0.1915	0.0556	0.2828	0.011
Diabetes	1 year	Base → TyG	0.0011	–0.0429	0.0345	0.768
Diabetes	1 year	TyG → TyG+EFV	0.0993	–0.0882	0.2889	0.191
Diabetes	2 years	Base → TyG	0.0342	–0.0092	0.0631	0.567
Diabetes	2 years	TyG → TyG+EFV	0.2499	0.1079	0.4076	0.005
Diabetes	3 years	Base → TyG	0.1571	0.0394	0.2914	0.040
Diabetes	3 years	TyG → TyG+EFV	0.3643	0.0811	0.6863	0.014

Base model: N-terminal pro–B-type natriuretic peptide+systolic blood pressure+diastolic blood pressure+estimated glomerular filtration rate+uric acid+left ventricular ejection fraction+New York Heart Association functional class+hypertension+coronary artery disease+atrial fibrillation.

**Table 9.** **IDI of different models adjusted by TyG and EFV for the incidence of primary endpoint at 1-, 2-, 3-year**.

Group	Time	Comparison	IDI_total	IDI_Lower	IDI_Upper	*p*
All	1 year	Base → TyG	0.0024	–0.0015	0.0063	0.229
All	1 year	TyG → TyG+EFV	0.0382	0.0130	0.0633	0.003
All	2 years	Base → TyG	0.0002	–0.0035	0.0053	0.692
All	2 years	TyG → TyG+EFV	0.0759	0.0381	0.1138	0.024
All	3 years	Base → TyG	0.0027	–0.0041	0.0095	0.441
All	3 years	TyG → TyG+EFV	0.1344	0.0688	0.2000	<0.001
Prediabetes	1 year	Base → TyG	0.0081	–0.0052	0.0214	0.233
Prediabetes	1 year	TyG → TyG+EFV	0.0320	–0.0004	0.0643	0.053
Prediabetes	2 years	Base → TyG	0.0005	–0.0141	0.0151	0.947
Prediabetes	2 years	TyG → TyG+EFV	0.0535	0.0092	0.0978	0.018
Prediabetes	3 years	Base → TyG	0.0112	–0.0105	0.0330	0.312
Prediabetes	3 years	TyG → TyG+EFV	0.0555	0.0146	0.1256	0.021
Diabetes	1 year	Base → TyG	–0.0021	–0.0077	0.0036	0.478
Diabetes	1 year	TyG → TyG+EFV	0.0434	–0.0018	0.0886	0.060
Diabetes	2 years	Base → TyG	–0.0011	–0.0065	0.0043	0.754
Diabetes	2 years	TyG → TyG+EFV	0.0897	0.0163	0.1631	0.017
Diabetes	3 years	Base → TyG	0.0013	–0.0049	0.0074	0.688
Diabetes	3 years	TyG → TyG+EFV	0.1911	0.0940	0.2883	<0.001

Base model: N-terminal pro–B-type natriuretic peptide+systolic blood pressure+diastolic blood pressure+estimated glomerular filtration rate+uric acid+left ventricular ejection fraction+New York Heart Association functional class+hypertension+coronary artery disease+atrial fibrillation.

## 4. Discussion

The principal finding of this study is that epicardial fat volume (EFV) and the triglyceride-glucose (TyG) index act as synergistic and incremental prognostic markers for primary endpoint in patients with CHF, particularly in the presence of glycemic dysregulation. To be noted, the prognostic power of EFV is gradient-dependent across the spectrum from normoglycemia to diabetes, being strongest in diabetes and significant in prediabetes. While a high TyG index alone significantly elevates the risk of primary endpoint, the highest risk is observed when both EFV and TyG are elevated. Also, EFV provides substantial incremental prognostic value beyond established risk factors and the TyG index, significantly improving model discrimination and reclassification.

Our results are consistent with and extend the current literature. Previous investigations have independently linked both EFV and the TyG index to adverse outcomes in various cardiovascular diseases [19,20]. However, our study is among the first to integrate them specifically in a HF cohort stratified by glycemic status. A crucial and novel aspect of our work is the highlighting of prediabetes as a critical risk stratum. Prediabetes is often under-managed in clinical practice [21,22]. Our data showed that patients with HF and prediabetes exhibit a clear intermediate phenotype, both in terms of baseline characteristics and prognostic marker performance, lying between normoglycemia and diabetes. The predictive accuracy of EFV in prediabetes was markedly stronger than in normoglycemic patients and approached the level seen in diabetes, indicating that the pathophysiological damage leading to adverse remodeling begins early in the dysglycemic continuum. This underscores the importance of early identification and intensified management in HF patients with even mild glycemic abnormalities, a population that may otherwise be overlooked [23,24,25].

Such synergistic relationship between EFV and the TyG index can be explained by their roles in the vicious cycle of metabolic-cardiac dysfunction. The TyG index, as a marker of systemic insulin resistance, promotes lipolysis and increases the flux of free fatty acids to ectopic fat depots, including the epicardium, thereby driving EFV expansion [26,27]. In the context of HF, this is particularly deleterious. A high EFV signifies a local source of pro-inflammatory cytokines (e.g., TNF-α, IL-6), adipokines, and free fatty acids, which can directly infiltrate the adjacent myocardium through paracrine and vasocrine mechanisms [28]. Notably, the adipokine profile from epicardial fat is often dysregulated in metabolic dysfunction—with increased secretion of pro-inflammatory adipokines (e.g., resistin, leptin) and reduced production of anti-inflammatory adipokines (e.g., adiponectin). This imbalance further perpetuates insulin resistance, promotes oxidative stress, and directly impairs myocardial contractility and energy metabolism, amplifying the local cardiac insult. Together with pro-inflammatory cytokines and free fatty acids, these adipokines exacerbate myocardial fibrosis, impair calcium handling, and promote endothelial dysfunction, leading to progressive ventricular remodeling and arrhythmogenesis [29,30,31]. Consequently, in diabetic HF patients, we observe the strongest correlation between TyG and EFV and the highest predictive power for primary endpoint, as both systemic and local metabolic insults are maximally activated.

The combined assessment of EFV and the TyG index can significantly improve risk prediction in heart failure patients, especially those with dysglycemia. This can help identify high-risk individuals who may benefit from more aggressive monitoring and therapy. Furthermore, EFV may represent a novel therapeutic target. Interventions aimed at reducing ectopic fat deposition, such as SGLT2 inhibitors or GLP-1 receptor agonists may exert part of their benefit by modulating epicardial fat [32]. Finally, clinicians should be vigilant about metabolic health in heart failure patients with prediabetes, as this group is already at significantly elevated risk.

This study demonstrated a powerful synergy between a marker of local cardiac metabolic burden (EFV) and a marker of systemic insulin resistance (TyG index) in predicting adverse outcomes in chronic HF. The risk is gradient-dependent on glycemic status, with a pronounced effect already evident in the often-neglected prediabetes population. The pathophysiological interplay between these markers creates a perfect storm that accelerates HF progression. The incorporation of EFV significantly refines risk prediction models beyond current standards.

## 5. Limitations

Several limitations of this study should be acknowledged. First, as a retrospective, single-center observational study, this study could not establish a definitive causal link between elevated EFV and endpoint. As a surrogate marker, EFV might reflect advanced metabolic exhaustion rather than a primary driver of adverse outcomes. Second, the diabetes subgroup (n = 112) was relatively moderate in size while the overall cohort was substantial. Meanwhile, the exclusive inclusion of patients who underwent CMR imaging might introduce selection bias. And although internal validation of the EFV cut-off was performed based on bootstrap resampling, the lack of external validation remained an important limitation of the present study. Baseline characteristics of the study population and inclusion criteria might introduce bias in the derived EFV cutoff value. Based on the research directions proposed in this study, findings regarding the EFV cutoff value require validation in larger, multicenter prospective cohorts to ensure broader generalizability, including subgroup analyses according to key clinical factors such as age and LVEF. Future studies will also aim to externally validate the EFV-TyG prognostic framework using independent datasets across different institutions, imaging protocols, and patient populations. Moreover, although we adjusted for numerous confounders, residual confounding might persist, particularly regarding clinical therapies. Finally, we recognized that EFV quantification based on CMR is relatively resource-intensive. The requirement for advanced imaging and specialized analyses will limit the immediate, widespread adoption of this framework in clinical settings with constrained resources.

## 6. Conclusion

Among patients with chronic heart failure, the combination of elevated epicardial fat volume and a high triglyceride-glucose index powerfully identifies those with glycemic dysregulation—including prediabetes and diabetes—who are at the greatest risk for clinical events. These findings provide a conceptual framework for integrated metabolic-cardiovascular risk stratification. Future research should focus on validating these markers in larger populations and exploring more cost-effective imaging alternatives to facilitate targeted metabolic interventions in this vulnerable population.

## Data Availability

Further information and requests for resources and reagents should be directed to and will be fulfilled by the lead contact, Prof. Xinli Li (xinli3267@njmu.edu.cn).
